# SDF1-Induced Antagonism of Axonal Repulsion Requires Multiple G-Protein Coupled Signaling Components That Work in Parallel

**DOI:** 10.1371/journal.pone.0018896

**Published:** 2011-04-27

**Authors:** E. Naomi Twery, Jonathan A. Raper

**Affiliations:** 1 Neuroscience Graduate Group, University of Pennsylvania School of Medicine, Philadelphia, Pennsylvania, United States of America; 2 Department of Neuroscience, University of Pennsylvania School of Medicine, Philadelphia, Pennsylvania, United States of America; National Institutes of Health (NIH), United States of America

## Abstract

SDF1 reduces the responsiveness of axonal growth cones to repellent guidance cues in a pertussis-toxin-sensitive, cAMP-dependent manner. Here, we show that SDF1's antirepellent effect can be blocked in embryonic chick dorsal root ganglia (DRGs) by expression of peptides or proteins inhibiting either Gα_i_, Gα_q_, or Gβγ. SDF1 antirepellent activity is also blocked by pharmacological inhibition of PLC, a common effector protein for Gα_q_. We also show that SDF1 antirepellent activity can be mimicked by overexpression of constitutively active Gα_i_, Gα_q_, or Gα_s_. These results suggest a model in which multiple G protein components cooperate to produce the cAMP levels required for SDF1 antirepellent activity.

## Introduction

The development of the nervous system requires the formation of numerous precise connections between neurons and their targets. Growth cones navigate through complex environments in which they are simultaneously exposed to many different guidance cues. Understanding how a growth cone integrates competing cues into a unitary guidance decision is a major challenge. One region of the developing nervous system in which axons are faced with competing guidance information is the developing optic nerve. For example, as axons leave the eye, they are simultaneously exposed to the potent repellent slit2 and to the chemokine SDF1, both of which are expressed along the optic stalk [1]-[5]. The presence of slit2 might be expected to preclude retinal extension, but SDF1 can mitigate its repellent effects. SDF1, acting through its G-protein coupled receptor CXCR4, has been shown to reduce the sensitivity of growth cones to a variety of repellents *in vitro* including slit2 [6].

The signaling pathway through which SDF1 reduces growth cone responses to repellents has been studied using wholly pharmacological approaches [6], [7]. SDF1's anti-repellent activity in primary neurons is blocked by pertussis toxin, which inhibits Gα_i_ or Gα_o_, and calmidazolium chloride, which inhibits calmodulin. SDF1 activity is also blocked by the PKA inhibitors PKI and Rp-cAMPs, and mimicked by the cAMP analogue Sp-cAMPs. Further, SDF1 activity is blocked by knockdown of the calcium/calmodulin-stimulated adenylate cyclase ADCY8 [8]. These findings suggest that increased cAMP levels are a component of the SDF1 antirepellent pathway, despite the apparent requirement for G proteins that canonically induce decreased cAMP levels. Although these studies provide an essential outline of the pathway, they leave many questions unanswered. One of these is how a pertussis toxin-sensitive pathway could lead to increased, rather than decreased, cAMP.

To better understand how CXCR4 activation increases cAMP levels, we began by investigating the identities of the G proteins required for antirepellent activity. We transfected primary neuronal cultures with constructs designed to block specific Gα or Gβγ subunits and assayed their effects on antirepellent signaling. Working downstream from these signaling components, we then examined the involvement of phospholipase C (PLC) in SDF1 signaling.

Here, we demonstrate that SDF1's antirepellent activity requires two distinct G alpha subunits, Gα_i_ and Gα_q_. We also show that anti-repellent signaling is abrogated by a Gβγ scavenger, GRK-CT. These results suggest that Gα_i_, Gα_q_, and Gβγ all cooperate to generate SDF1 antirepellent activity. We also show that antirepellent signaling is blocked by PLC inhibitors. Taken together with previous findings, these results are consistent with SDF1/CXCR4 signaling acting through multiple G protein subunits that work together to activate PLC, which in turn ultimately leads to elevated internal calcium levels that stimulate the calcium/calmodulin-dependent adenylate cyclase ADCY8 to produce cAMP.

## Materials and Methods

### Ethics statement

Chick embryos were maintained according to University of Pennsylvania Institutional Animal Care and Use Committee (IACUC) guidelines, approved as protocol #802243.

### Cell culture and explant-based collapse assays

Fertile chicken eggs were purchased from B&E Eggs, York Springs, PA. DRGs were dissected from E7 chick embryos and grown on laminin-coated coverslips in F12 supplemented medium as previously described [1]. Explants were cultured for 18-20 hours before treatment. SDF1 (50 nM, Invitrogen), supernatant from sema3A-transfected 293T cells, and/or pharmacological inhibitors as noted were added to wells at the same time. Cells were returned to the incubator for 30 minutes and then fixed for at least 30 minutes with 4% paraformaldehyde plus 10% sucrose in PBS. Growth cones were examined on an Axiovert 35 (Zeiss) with phase optics and scored as collapsed if they had no lamella and no more than two filopodia as described in [9]. Numbers of collapsed and uncollapsed growth cones from pairs of treatment conditions were compared with a two-tailed Fisher Exact Test and considered significant if p<0.05. Statistical comparisons were performed with Prism (GraphPad, La Jolla, CA).

### Transfection

E7 chick DRGs were dissociated by incubation with 0.25% trypsin-EDTA (Invitrogen) for 20 minutes at 37°C and then resuspended in Amaxa nucleofector solution. Cells from 12 ganglia were electroporated with 4 µg total plasmid DNA using the G-013 program for the rat neuron kit and the Amaxa nucleofector (Lonza). Plasmid volume varied from 3–10 µL, depending on plasmid concentration. Cells were cotransfected with EYFP or Citrine (2 µg) and an experimental plasmid (2 µg). Transfected cells were cultured as described above for 24 hours before treatment with sema3A supernatant. Plasmid-expressing cells were identified by expression of EYFP or Citrine and counts of brightly green growth cones were analyzed as above.

### Plasmids and Reagents

Expression plasmids for constitutively active G proteins, RGS proteins, and dominant-negative Gα_i_ were obtained from the Missouri Science and Technology cDNA Resource Center (Rolla; cdna.org). An expression plasmid containing GRK-CT was provided by P. Alberts [10]. Expression plasmids encoding G protein interfering peptides were obtained from Cue Biotech [11]. The PLC inhibitor U73122 (Sigma) was used at 20nM.

### Immunostaining

Fixed cultures were washed once with PBS and 3 times with PBS + 0.1% Triton-X100, then blocked for half an hour in blocking reagent: PBS + 3% bovine albumin, 1% PVP-10, 1% PVP-40, and 0.1% PVP-360 (Sigma) with 0.2% Triton-X100 added. Goat anti-GFP (Rockland) or mouse anti-HA (Covance) were used at 1∶500 and visualized with AlexaFluor secondary antibodies (Invitrogen). Cultured cells were imaged either on a Zeiss Axiovert 35 with a 63× objective or on a (Leica Confocal) with a 63× objective and 3× zoom. Multiple colors were imaged with line-by-line sequential scanning.

## Results

### Blocking Gα_i_ or Gα_q_ blocks SDF1 antirepellent activity

Semaphorin 3A (sema3A) is a powerful repellent for dorsal root ganglion (DRG) axons [12]. Bath application of sema3A to DRG growth cones induces them to transition from a spread motile morphology to a collapsed shape without lamellae and few filopodia (Luo et al., 1993). This dramatic change in morphology can be used to measure the strength of repellent cues or to measure the relative susceptibility of growth cones to repellent cues. Using this assay, the repellent responses of DRG growth cones to sema3A, sympathetic growth cones to sema3C, or retinal growth cones to slit2, have all been shown to be greatly reduced in the presence of the chemokine SDF1 [6]. SDF1 by itself has little discernible effect on these growth cones, but when SDF1 is present, 5 to 8 times more repellent is required to induce half maximal growth cone collapse [6].

SDF1 acts through its seven transmembrane receptor, CXCR4, to mitigate the ability of repellents to collapse growth cones [6]. Paradoxically, although its signaling pathway in primary neurons is blocked by the Gα_i/o_ blocker pertussis toxin [6], SDF1 appears to induce increased cAMP levels. Previous work from our laboratory showed that SDF1's antirepellent effects can be blocked by the cAMP antagonist RpcAMPs or mimicked by the cAMP analogue SpcAMPs [6]. An SDF1-induced rise in cAMP has been observed in cultured primary chick retinal neurons [8]. To better define the specific G-protein components through which SDF1 acts, dissociated DRGs were co-transfected with expression constructs for EYFP along with plasmids encoding short peptides that selectively block signaling through specific Gα containing G-proteins. These peptides are derived from the C termini of the Gα proteins they target and they selectively compete with the targeted Gα proteins for receptor binding [11]. Their selectivity and effectiveness has been demonstrated in several other systems, including zebrafish [13] and fly [14].

**Figure 1:**
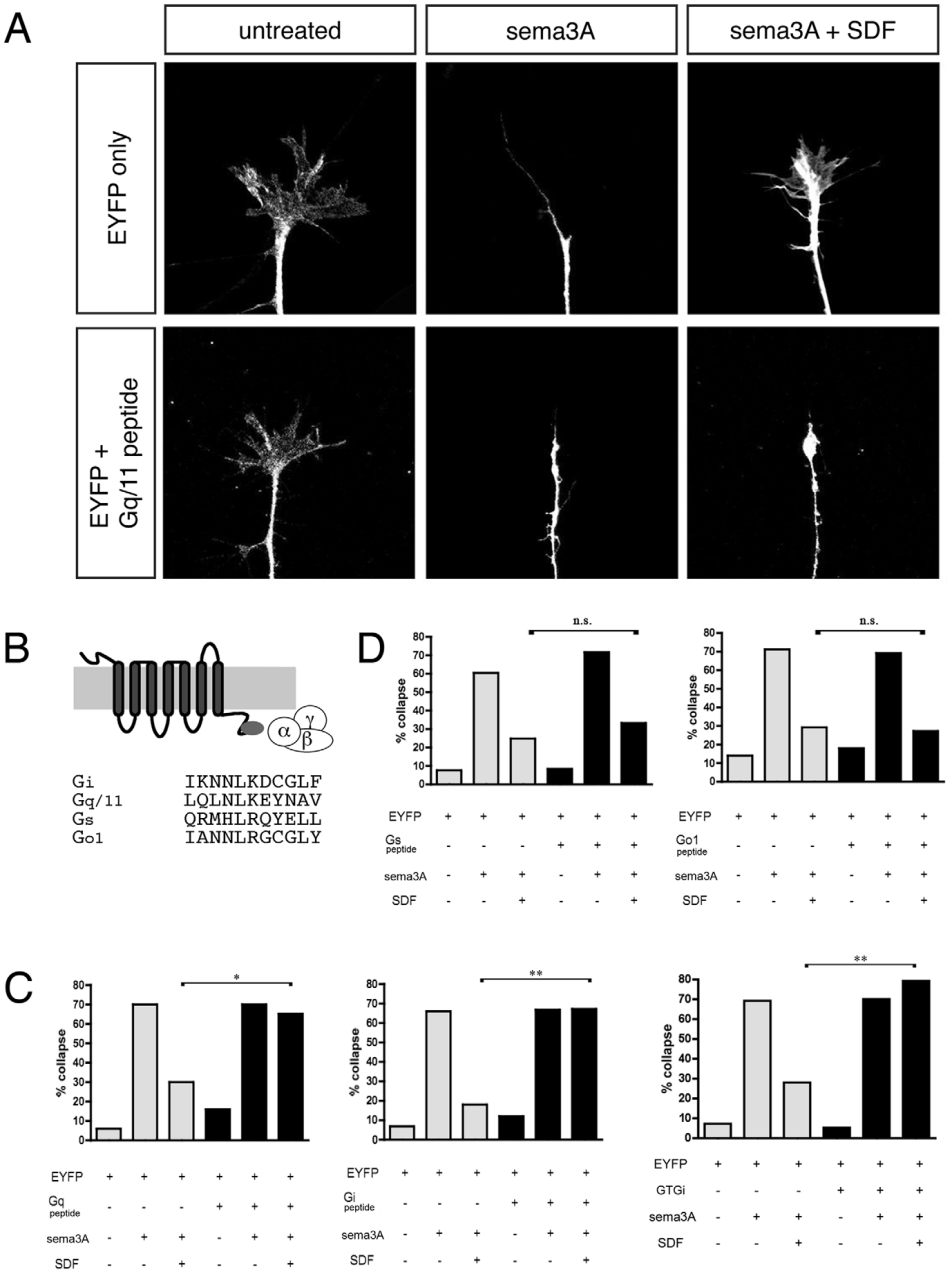
Competitive inhibitors of Gα_i_ or Gα_q/11_, but not Gα_s_ or Gα_o_, block SDF1 mediated antirepellent activity. (**A**) Growth cones of dissociated DRGs transfected with EYFP or EYFP + Gq/11 inhibitory peptide have motile lamellae and filopodia. (**B**) Specific inhibitory Gα peptides (medium grey) bind selected GPCRs and prevent their association with functional G proteins containing the same Gα peptide sequence. (**C,D**) Dissociated DRGs were transfected with EYFP-only (grey bars) or with EYFP and an experimental construct (black bars). After 24 h in culture, cells were treated for 30' with sema3A or with sema3A + SDF1. (**C**) The SDF1 antirepellent response is blocked by a by peptides targeting Gα_i_ or Gα_q/11_, and also by a full-length dominant-negative Gα_i_. (**D**) The SDF1 antirepellent response is not affected by peptides targeting Gα_s_ or Gα_o_. *, p<0.001; **, p<0.0001.

DRG neurons transfected with EYFP alone collapse in response to sema3A (Fig. 1A; compare the first and second grey bars in Fig. 1C, D). The presence of SDF1 makes DRG growth cones resistant to sema3A (Fig. 1A; compare second and third grey bars in Fig.1C, D). For these experiments, transfected DRG cultures were stained for EYFP and only those growth cones that were brightly fluorescent were counted. In EYFP-only conditions, cultures show low background collapse. The percentage of collapsed growth cones increases in the presence of sema3A but increases significantly less when SDF1 is added along with sema3A. Co-transfection of expression plasmids encoding EYFP along with peptides targeting Gα_q/11_ (Fig. 1C, first panel) or Gα_i1/2_ (Fig. 1C, second panel) have no effect upon DRG growth cone collapse in the presence of sema3A alone (compare the middle grey bars to the middle black bars). However, the Gα_q/11_ or Gα_i1/2_ peptides do block SDF1's ability to reduce collapse in response to sema3A (compare the third grey bars to the third black bars). This suggests that both Gα_q_ and Gα_i_ mediated G-protein coupled signaling are each required for SDF1's antirepellent effect. A full-length dominant negative Gα_i_ that has been shown to be effective in transfected CHO cells [15] was tested for its ability to block SDF1-mediated signaling. This construct also blocked the SDF1 antirepellent effect, corroborating the finding with the Gα_i_ based peptide (Fig. 1C, third panel). Co-transfection of EYFP with peptides targeting Gα_s_ or Gα_o1_ had no effect on DRG responses to sema3A or to SDF1 (Fig. 1D). Because the effectiveness of the Gα_s_ and Gα_o1_ peptides have been tested in other systems [16], [17], the Gα_i1/2_ and Gα_q_ peptides were effective, and all interfering peptides were expressed from identical expression plasmids, we conclude that Gα_s_ and Gα_o_ are unlikely to be required for antirepellent activity.

### Both Gβγ and Gα are necessary for SDF1 antirepellent activity

**Figure 2:**
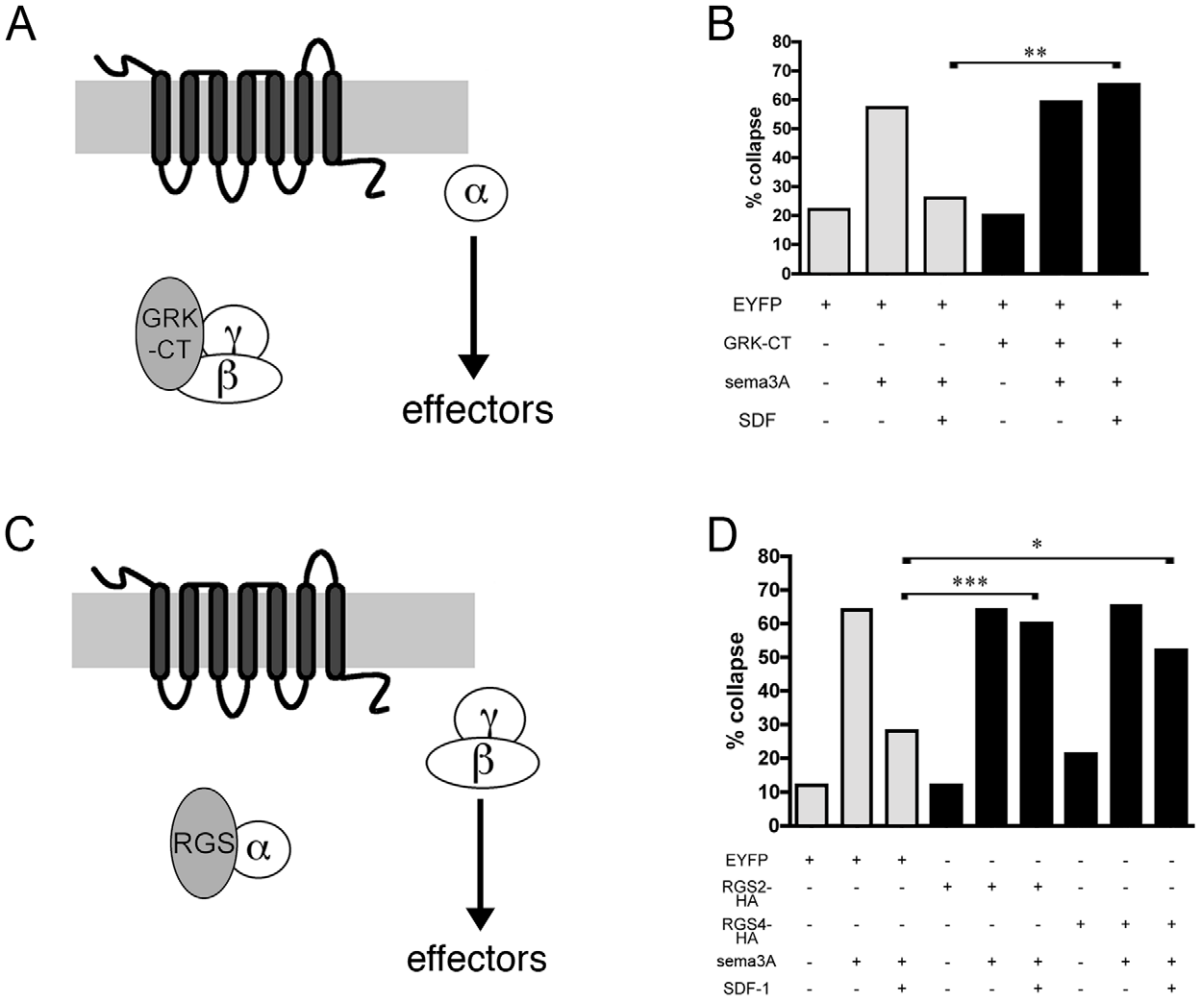
Scavengers of Gβγ, Gα_i_, or Gα_q_ subunits block SDF1 antirepellent activity. (**A**) GRK-CT sequesters βγ subunits while leaving α free to activate downstream effectors. (**B**) Transfection of dissociated DRGs with GRK-CT blocks the SDF1 antirepellent effect but does not alter background collapse or response to sema3A. (**C**) RGS proteins sequester specific α subunits and hasten their inactivation while leaving βγ subunits free to activate downstream effectors. (**D**) Transfection of dissociated DRGs with either RGS2, a α_q_ specific GAP, or RGS4, an α_i_ and to a lesser extent α_q_ specific GAP, block SDF1 antirepellent activity without affecting background collapse or response to sema3A. *, p<0.05; **, p<0.005; ***, p<0.001.

Because the short inhibitory peptides we used block the initial receptor mediated dissociation and activation of G proteins, they cannot determine whether SDF1 signaling depends upon alpha or beta-gamma subunits to activate downstream targets. We used the C-terminal portion of GRK2, or GRK-CT, as a Gβγ scavenger that should prevent the complex from stimulating downstream targets (Fig. 2A). Ghahremani et al. [10] showed that this protein fragment could block Gβγ-specific calcium release in LD2S cells and this construct has since been widely used. Coexpression of GRK-CT with EYFP does not increase background collapse or interfere with growth cones' responses to sema3A (Fig. 2B). GRK-CT does, however, block SDF1-induced reduction in sema3A-mediated growth cone collapse, suggesting that SDF1 antirepellent activity requires Gβγ-induced activation of downstream targets.

We next set out to determine whether specific Gα subunits activate downstream targets in SDF1 mediated antirepellent signaling. RGS proteins act as GAPs for Gα subunits (Fig 2C). RGS2 specifically binds and inactivates Gα_q_, and RGS4 primarily binds Gα_i_ but also binds Gα_q_ to a lesser extent [18], [19]. Coexpression of either RGS2 or RGS4 with EYFP does not affect background levels of collapse, nor does it interfere with sema3A induced collapse (Fig. 2D). Expression of either RGS2 or RGS4 does however, interfere with SDF1's ability to reduce collapse in response to sema3A (Fig. 2D). These results suggest that Gα_q_, and possibly Gα_i_, activate downstream targets in the SDF1 mediated antirepellent pathway.

### Constitutively active Gα subunits

**Figure 3:**
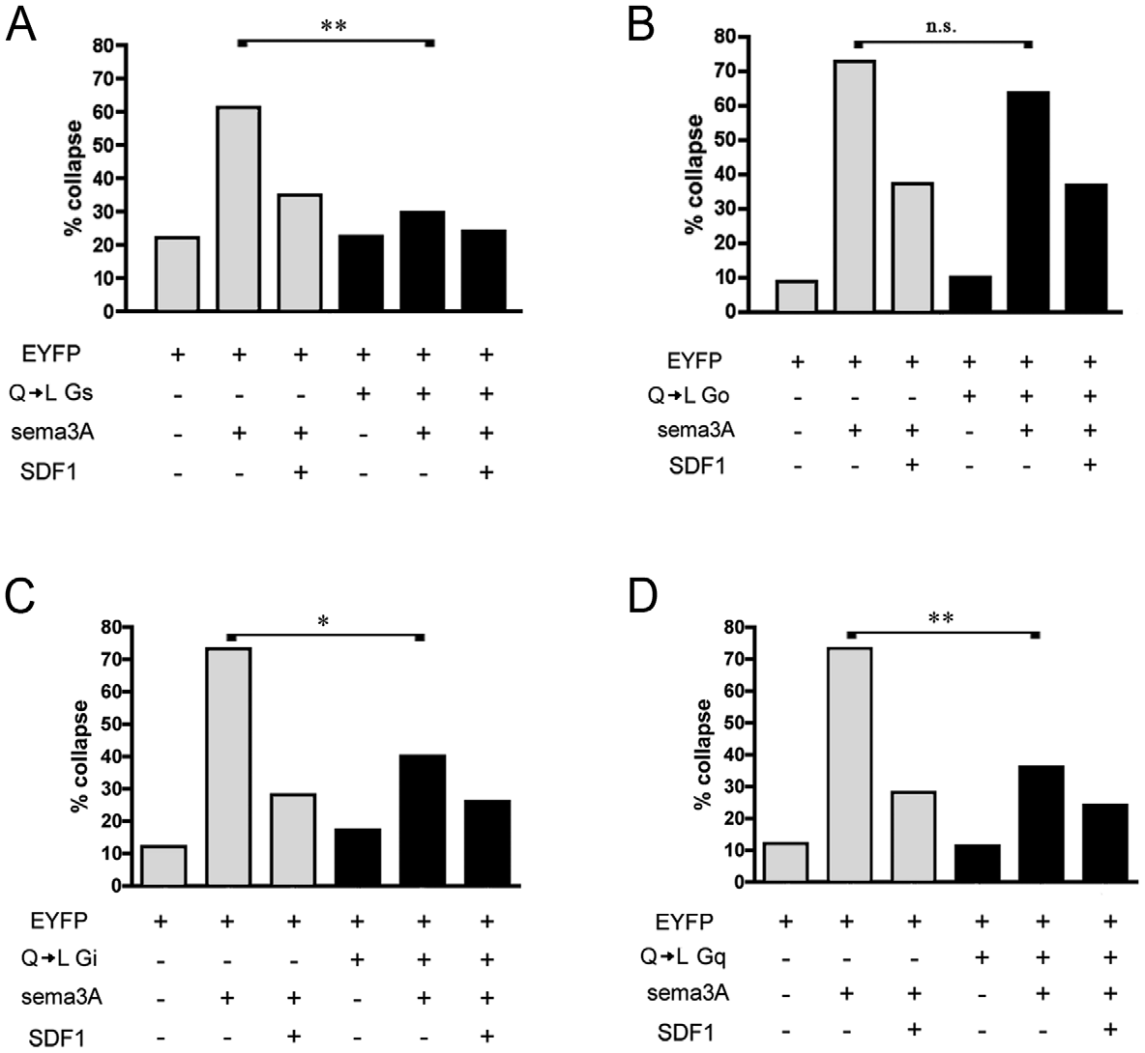
Constitutively active Gα_q_, Gα_i_, or Gα_s_ mimic SDF1's antirepellent effect. (**A**) Transfection of QL Gα_s_ into DRGs makes them unresponsive to sema3A. (**B**) Transfection of QL Gα_o_ into DRGs has no effect on their responses to sema3A or SDF1. (**C**) Transfection of DRGs with QL Gα_i_ or with (**D**) QL Gα_q_ makes DRGs unresponsive to sema3A. *, p<0.001, **, p<0.0001.

We next asked whether overexpression of specific constitutively active Gα subunits can mimic SDF1 induced antirepellent activity. Gα subunits with a Q to L mutation in the nucleotide-binding region are unable to cleave GTP and are thereby made constitutively active [20]-[23]. Coexpression of QL Gα_s_ with EYFP made DRG growth cones insensitive to sema3A in a manner similar to SDF1, and what is more, SDF1 induced little additional antirepellent effect (Fig. 3A). Similar results were obtained with constitutively active Gα_i_ (Fig. 3C) or QL Gα_q_ (Fig. 3D). QL Gα_o_ had no effect on growth cone responses to either sema3A or SDF1 (Fig. 3B). These results suggest that Gα_s_, Gα_q_, or Gα_i_ each individually have the capability of initiating signaling events similar to those induced by SDF1, whether or not they participate in SDF1 signaling under normal circumstances.

### Inhibition of phospholipase C blocks SDF1 antirepellent activity

**Figure 4:**
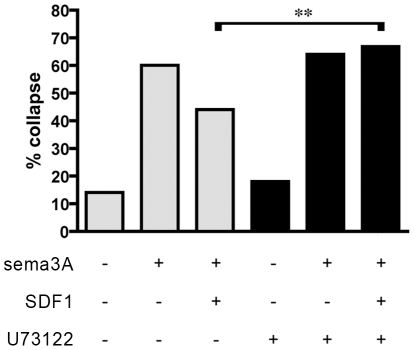
Inhibiting PLC blocks SDF1 antirepellent activity. DRG explants were treated with 20 nM PLC inhibitor U73122 (black bars). U73122 does not alter background collapse or DRG responsiveness to sema3A, but does block the antirepellent effect of SDF1. **, p<0.0001.

Because phospholipase C (PLC) is a classical effector of Gα_q/11_-class G proteins [24], [25], and since our results show a requirement for Gα_q/11_ activity in SDF1 mediated antirepellent signaling, we hypothesized that PLC is required for SDF1's antirepellent activity. The PLC inhibitor U73122 has no effect on background collapse or on growth cone responsiveness to sema3A (Fig. 4, grey bars). U73122 does, however, block SDF1's ability to reduce growth cone responses to sema3A (Fig. 4, black bars).

### Inhibition of phospholipase C blocks antirepellent effects induced by constitutive Gα_q_ activity

**Figure 5:**
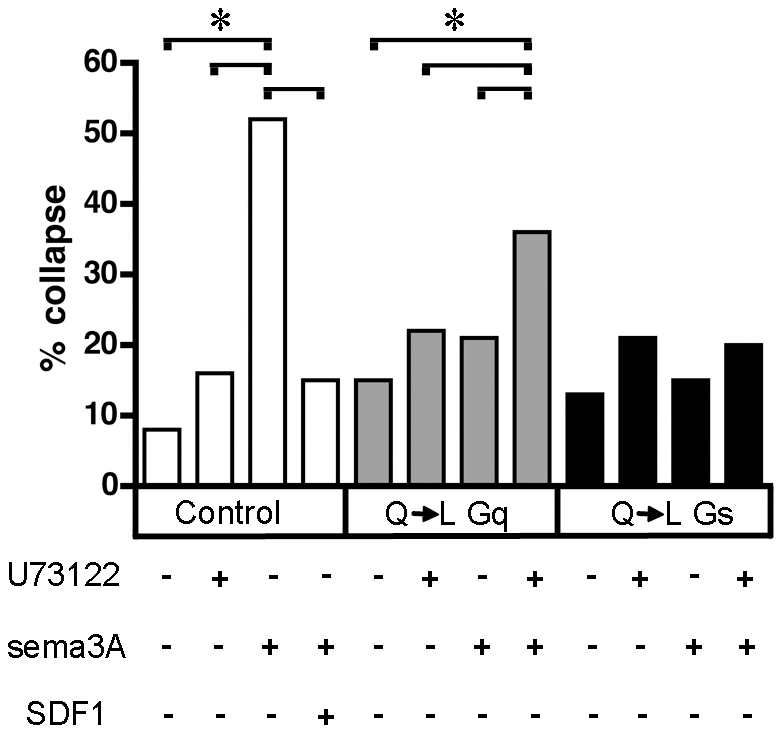
Inhibiting PLC blocks antirepellent activity induced by expression of a constitutively active Gα_q_. DRGs were transfected with expression plasmids for Citrine (control, empty bars), Citrine and QL Gα_q_ (grey bars), or Citrine and QL Gα_s_ (black bars). Expression of QL Gα_q_ makes growth cones insensitive to sema3A unless the PLC blocker U73122 (20 nM) is also present. Growth cones expressing QL Gα_s_ are insensitive to sema3A in both the absence and the presence of U73122. *, p<0.002.

We next tested whether Gα_q_ activation can induce an anti-repellent response through the activation of PLC. As already demonstrated, sensory axons expressing a control Citrine construct collapse in response to sema3A and this collapse is largely mitigated in the presence of SDF1 (Figure 5, empty bars). In contrast, growth cones expressing the constitutively active QL Gα_q_ are insensitive to sema3A. (Figure 5, grey bars). Significant sensitivity to sema3A is restored, however, when PLC is blocked. Growth cones expressing QL Gα_s_ are also insensitive to sema3A, but this insensitivity is not reversed by blocking PLC (Figure 5, black bars). These findings are consistent with the idea that SDF1 induced antirepellent activity is mediated by Gα_q_ activation of PLC, while constitutively active Gα_s_ mediated antirepellent activity is not. As discussed in more detail below, one attractive explanation for these observations is that SDF1 induced activation of PLC indirectly induces elevated cAMP levels through a separate mechanism from the more traditional direct activation of adenylate cyclases by Gα_s_.

## Discussion

Although G protein coupled receptors (GPCRs) are often pictured as acting through specific, dedicated G proteins, it is now known that a single GPCR can bind and activate G proteins from more than one G alpha class [see 26]. PAR1, a thrombin receptor, can bind to Gα_i/o_, Gα_q_, or to Gα_12/13_ [27]. β2-adrenergic receptors, when phosphorylated by PKA, switch affinities from Gα_s_ to Gα_i_ [28]. The class I metabotropic glutamate receptor mGluR1 has been shown to bind Gα_i/o_, Gα_q/11_, and Gα_s_, at least in certain cell types [29]. Wang et al. [30] found that parathyroid hormone receptor 1 regulates different genes with different G proteins or combinations of G proteins, suggesting that individual G proteins might be required for some cell behaviors but not for others. These are just a few of the many examples of GPCRs coupling to multiple G proteins.

Chemokine receptors as a class are generally thought to signal through Gα_i/o_-type G proteins to decrease cAMP, activate PI3K, and activate both p38 and ERK1/2 MAP Kinases (see [31], [32] for reviews). PI3K activation leads to activation of a number of other kinases, including Akt. SDF1 signaling through the chemokine receptor CXCR4 is also associated with changes in transcription, usually mediated through MAPK or Akt, that contribute to cell survival [4], [33]–[35]. However, several groups have found that CXCR4 signals through other classes of G proteins. Maghazachi [36] reported that antibodies targeting Gα_o_ or Gα_q_, but not Gα_i_, Gα_s_, or Gα_z_, could block SDF1-induced chemotaxis in natural killer cells. Soede et al. [37] found that CXCR4-dependent migration of myeloid leukemia cells require either the combination of Gαi and Gα_q_ or Gα_q_ alone, depending on the destination tissue. Tan et al. [38] showed that SDF1/CXCR4-induced migration of Jurkat T cells required both Gα_13_, which activated Rho, and Gα_i_. These and other studies raised the possibility that SDF1/CXCR4 signaling in axon guidance might be more complex than that of the classic chemokine signaling pathway. Previous work from our laboratory [6] identified several components of SDF1/CXCR4 signaling in the antirepellent pathway, including a pertussis toxin-sensitive G protein, increased cAMP, and activation of PKA. In addition to the surprising apparent increase in cAMP levels observed in these previous studies, the effects of SDF1 on axonal responses to repellents were found to be independent of PI3K/Akt signaling and of MAPK.

The findings in this study show that SDF1's antirepellent activity can be blocked separately by Gα_i_, Gα_q/11_, or Gβγspecific competitive inhibitors. These data suggest that each is required for the normal function of the antirepellent pathway. However, we also found that overexpression of constitutively active forms of Gα_i_ or of Gα_q_ can mimic application of SDF1. This suggests that either one of these signaling components is capable of stimulating a common downstream element that is sufficient for activation of the pathway. These findings are consistent with the idea that SDF1 stimulates multiple G protein coupled pathways to a degree that is insufficient for any one of them alone to induce a physiological response, but in combination, their actions sum to a level above a threshold for activation to produce an antirepellent response.

**Figure 6:**
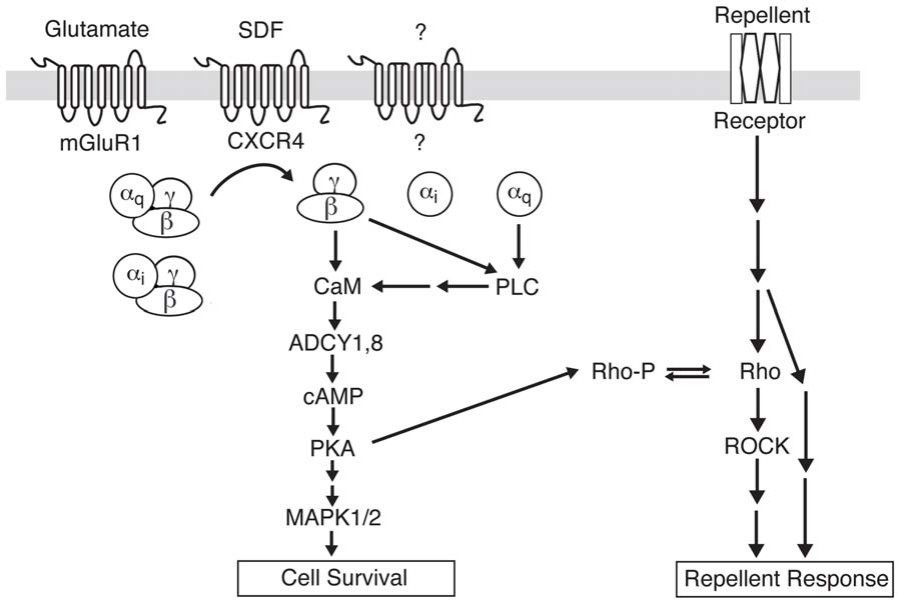
A model for antirepellent signaling. We identify roles for Gα_i_, Gα_q_, and Gβγ, as well as PLC, in the antirepellent response to SDF1. Previous work has shown requirements for calmodulin, ADCY8, cAMP, and PKA, along with inhibition of Rho and ROCK. In our model, CXCR4 activates Gα_i_ and Gα_q_, and they and their associated Gβγ subunits cooperate to activate PLC. PLC, through generation of diacylglycerol and inositol trisphosphate (Taylor et al., 1991), increases calcium levels. Increased calcium activates calmodulin, which in turn activates ADCY8 and thereby increases cAMP. Increased cAMP activates PKA, which phosphorylates MAPK1/2, leading to its activation and function in cell survival. PKA also phosphorylates Rho, which is thereby inactivated. This inactivation and the subsequent inactivation of ROCK are required for the antirepellent response to SDF1.

We also found that overexpression of a constitutively active Gα_s_ can mimic SDF1 even though a competitive inhibitor of Gα_s_ does not block SDF1 mediated signaling. As Gα_s_ is a canonical stimulator of adenylate cyclase activity and would be expected to elevate cAMP levels, this finding is consistent with the idea that the common element upon which Gα_i_, Gα_q_, and Gβγ all converge downstream from SDF1 activation of CXCR4 is elevated cAMP levels. Thus, our proposed model of the signaling pathway is that Gα_i_, Gα_q_, and their associated βγ subunits all cooperate to increase the local concentration of cAMP, leading to suppression of axonal repulsion (Fig. 6). The ability of Gα_s_ to accomplish the same thing through a different route raises the possibility that a very wide range of GPCRs could influence axonal responses to repellents and axonal pathfinding.

Previous work has shown that SDF1's antirepellent activity requires calmodulin and the calcium/calmodulin-stimulated cyclase ADCY8 [6], [8]. Xu [8] also showed by Förster resonance energy transfer (FRET) that SDF1 stimulates increased cAMP levels, and that this can be blocked by inhibition of calmodulin. Gα_i_ and Gα_q_ are not ordinarily associated with increases in cAMP, yet our results show that they are required components in the antirepellent signaling pathway. Gα_q_ and Gβγ activity, through the activation of PLC, can produce diacylglycerol and inositol trisphosphate and thereby increase intracellular calcium [39]. Thus, our present finding that both Gα_q/11_ and PLC are required for SDF1 antirepellent activity provides a connection between the G proteins activated by SDF1 and the calmodulin and calcium/calmodulin-stimulated cyclase that has been shown to increase cAMP downstream of SDF1. Our results are consistent with a signaling pathway (Fig. 6) in which multiple G protein components stimulate PLC activity that induces an increase in intracellular calcium levels and leads to the activation of calmodulin. Calmodulin, in turn, activates calcium/calmodulin-stimulated adenylate cyclases, such as ADCY8, and thereby increases cAMP.

Some of the important questions that remain include how elevated cAMP levels decrease growth cone responses to repellents and the degree to which this modulation of repellent effectiveness is important in axonal pathfinding *in vivo*. Both SDF1/CXCR4 activity and activity of the calmodulin-activated adenylate cyclases have a strong influence on axonal responses to the repellent slit *in vivo* [8]. Our findings in this study suggest that activation of a wide range of GPCRs that signal through Gα_i_, Gα_q_, or Gα_s_ could potentially participate in axon guidance decisions.
